# Synthesis of (3*R*,5*R*)-harzialactone A and its (3*R*,5*S*)-isomer

**DOI:** 10.3762/bjoc.6.8

**Published:** 2010-01-29

**Authors:** Gowravaram Sabitha, Rangavajjula Srinivas, Sukant K Das, Jhillu S Yadav

**Affiliations:** 1 Organic Division I, Indian Institute of Chemical Technology, Hyderabad 500 007, India, Fax: +91-40-27160512

**Keywords:** dithiane, harzialactone A, hydroxyl directed reduction, stereoisomer

## Abstract

The total synthesis of (3*R*,5*R*)-harzialactone A (**1**) and its (3*R*,5*S*)-isomer (**2**) is described. Epoxide opening with thioacetal and diastereoselective reductions are used as key reactions.

## Introduction

**Figure 1:**
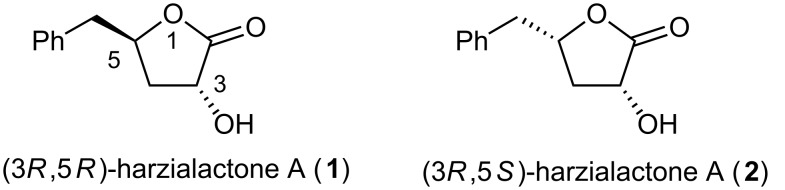
Natural harzialactone A (**1**), and its (3*R*,5*S*)-isomer (**2**).

Marine microorganisms such as bacteria, fungi, and microalgae have proved to be a rich source of structurally novel and biologically active secondary metabolites [1]. (+)-Harzialactone A (**1**), a marine metabolite isolated from the culture broth of a strain of *Trichoderma harzianum* OUPS-N115 by Numata and co-workers, exhibited antitumor and cytotoxic activities against cultured P388 cells [2]. The absolute configuration of (+)-**1** was established based on ^1^H NMR studies and by its synthesis [3–4]. Harzialactone A (**1**) (Figure 1) is a synthetic target of considerable interest due to its potent biological activity and unique structure. A few methods for its synthesis have been documented in the literature [3–10] as well as a synthesis of nonnatural (−)-harzialactone A [11]. However, the anti-tumor activity of Harzialactone A coupled with its unique structural architecture prompted us to attempt its synthesis.

**Scheme 1:**
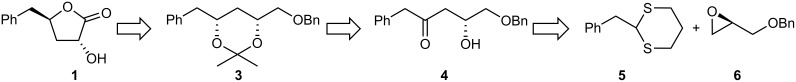
Retrosynthesis of harzialactone A (**1**).

The retrosynthesis is depicted in Scheme 1. Harzialactone **1** could be made from **3** by successive protecting group transformations. **3** can be made by hydroxyl directed reduction of **4** which in turn could be prepared by epoxide **6** opening with dithiane **5**.

## Results and Discussion

**Scheme 2:**
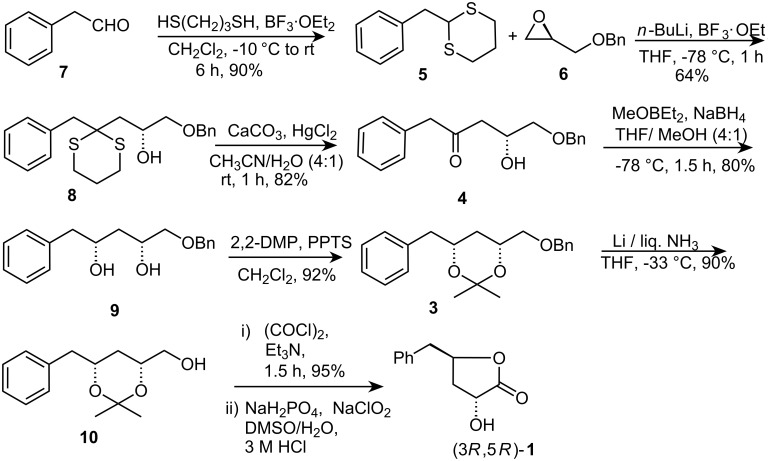
Synthesis of natural harzialactone A (**1**).

The synthesis of natural (3*R*,5*R*)-**1** was initiated from the known epoxide **6**, which is commercially available. Treatment of 2-phenylacetaldehyde **7** with 1,3-propanedithiol in the presence of BF_3_·Et_2_O in CH_2_Cl_2_ afforded thioacetal **5** in 90% yield (Scheme 2). The epoxide **6** was coupled with the acyl anion equivalent **5** (1.0 equiv), prepared by metallation at –78 °C with 1.0 equiv of *n*-butyllithium in the presence of BF_3_·Et_2_O to obtain **8** in 64% yield. Removal of the dithioketal using HgCl_2_/CaCO_3_ in CH_3_CN/H_2_O (4:1)[12] provided the corresponding hydroxyketone **4** in 82% yield. Treatment of **5** with NaBH_4_ and MeOBEt_2_ [13–14] stereoselectively formed the *syn* diol **9** in good yield (80%). The diol **9** was subsequently transformed into the isopropylidene derivative **3** by treatment with 2,2-dimethoxypropane and a catalytic amount of PPTS in CH_2_Cl_2_.

In the ^13^C NMR spectrum of **3**, the acetonide methyl groups resonated at 19.6 and 29.9 ppm indicating a 1,3-*syn*-relationship that was further substantiated by the appearance of the quaternary carbon in the downfield region (98.7 ppm). Deprotection of the benzyl group using Li/liq. NH_3_ gave alcohol **10**. Oxidation of alcohol **10** under Swern conditions and further oxidation of the resulting aldehyde using NaH_2_PO_4_, NaClO_2_ in DMSO/H_2_O furnished the target hydroxylactone (3*R*,5*R*)-**1** as reported earlier. The IR absorption at 1774 cm^−1^ indicates the presence of δ-lactone system.

**Scheme 3:**
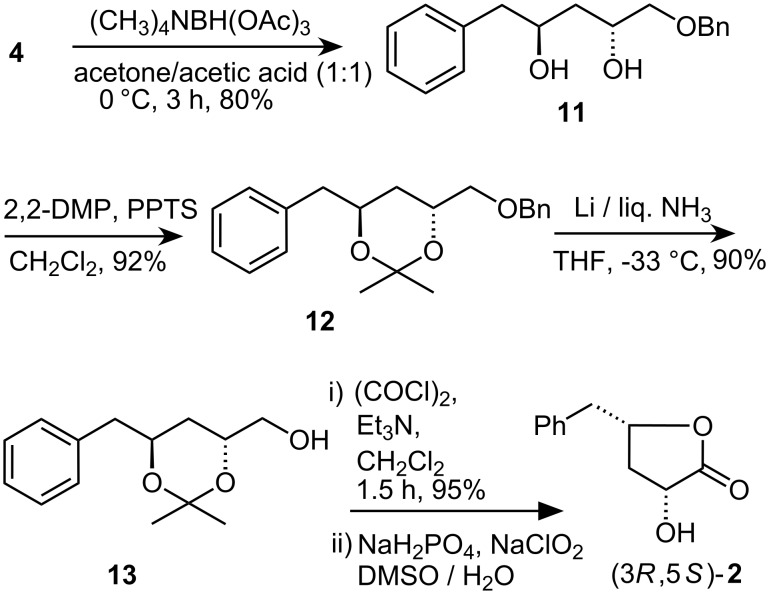
Synthesis of (3*R*,5*S*)-harzialactone A (**2**).

The synthesis of (3*R*,5*S*)-**2** was also accomplished in an identical manner from **4** (Scheme 3). The substrate hydroxyl directed asymmetric reduction with Me_4_NBH(OAc)_3_ [15–16] was performed at 0 °C to afford the *anti* diol **11** as the major product, which was converted into stereoisomer (3*R*,5*S*)-**2** via acetonide **12**, deprotection of benzyl group to give **13**, and further functional group transformations by use of the same reagents and conditions as those described for the conversion of **10** into **1**. The IR absorption at 1775 cm^−1^ confirms the presence of δ-lactone in (3*R*,5*S*)-**2**.

The *anti* relationship of two hydroxyl groups was studied in compound **12**. In the ^13^C NMR of **12**, the acetonide methyl groups resonated at 24.9 and 34.2 ppm indicating a 1,3-*anti*-relationship that was further substantiated by the appearance of the quaternary carbon in the downfield region (100.5 ppm) [7].

In conclusion, a stereoselective synthesis of natural (+)-(3*R*,5*R*)-harzialactone A and its nonnatural stereoisomer (3*R*,5*S*) has been accomplished.

## Supporting Information

File 1 Experimental section and analytical data.
