# Supplementary material for: Synthesis of (3R,5R)-harzialactone A and its (3R,5S)-isomer
Source: Beilstein J Org Chem. 2010 Jan 29;6:8. doi: 10.3762/bjoc.6.8 (PMC2871370; doi:10.3762/bjoc.6.8)

**Supporting Information**

**to**

**Synthesis of (3*R*,5*R*)-harzialactone A and its (3*R*,5*S*)-isomer**

Gowravaram Sabitha,^*^ Rangavajjula Srinivas, Sukant K Das and Jhillu S. Yadav

Address: Organic Division I, Indian Institute of Chemical Technology, Hyderabad 500 007, India, Fax: +91-40-27160512

Email: Gowravaram Sabitha*- [gowravaramsr@yahoo.com](mailto:gowravaramsr@yahoo.com)

*Corresponding Author

**Experimental**

**General**

Reactions were conducted under N_2_ in anhyd solvents such as CH_2_Cl_2_, THF and EtOAc. All reactions were monitored by TLC (silica-coated plates and visualizing under UV light). *n*-Hexane (bp 60–80 °C) was used. Yields refer to chromatographically and spectroscopically (^1^H and ^13^C NMR) homogeneous material. Air sensitive reagents were transferred by syringe or double-ended needle. Evaporation of solvents was performed at reduced pressure on a Buchi rotary evaporator. ^1^H and ^13^C NMR spectra of samples in CDCl_3_ were recorded on Varian FT-200MHz (Gemini) and Bruker UXNMR FT-300 MHz (Avance) spectrometers. Chemical shift δ is reported relative to TMS (δ = 0.0) as an internal standard. Mass spectra were recorded E1 conditions at 70 eV on LC-MSD (Agilent technologies) spectrometers. All high resolution spectra were recorded on QSTAR XL hybrid MS/MS system (Applied Biosystems/MDS sciex, foster city, USA), equipped with an ESI source (IICT, Hyderabad). Column chromatography was performed on silica gel (60-120 mesh) supplied by Acme Chemical Co., India. TLC was performed on Merck 60 F-254 silica gel plates. Optical rotations were measured with a JASCO DIP-370 Polarimeter at 20 °C. The % ee of the products was determined using Chiral HPLC, DIONEX (chromeleon, PDA).

**2-Benzyl-1,3-dithiane (5):**

To a stirred solution of phenyl acetaldehyde **7** (5 g, 41.66 mmol) and 1,3-propane dithiol (4.6 mL 45.82 mmol) in dry CH_2_Cl_2_ (30 mL) at -10 °C under nitrogen atmosphere was added BF_3_.OEt_2_ (0.5 mL, 4.16 mmol) and allowed to warm to room temperature. After stirring for 6 h at room temperature, the reaction mixture was cooled to 0 °C, quenched with 2 M NaOH solution (20 mL), and extracted with CH_2_Cl_2_ (3 × 20 mL). The combined organic extracts were washed with H_2_O (1 × 20 mL) and brine (1×10 mL), and dried over anhydrous Na_2_SO_4_. Evaporation of the solvent and purification of the residue by silica gel column chromatography using 2% EtOAc-hexane as eluent afforded **5** as a colorless viscous liquid.

Yield: (7.87 g, 90%), IR (KBr): 3027, 2935, 2898 cm^-1^.

^1^H NMR (CDCl_3_, 300 MHz): δ =7.35-7.22 (m, 5H), 4.25 (t, *J* = 7.3 Hz, 1H), 3.02 (d, *J* = 7.3 Hz, 2H), 2.86-2.80 (m, 4H), 2.17-2.06 (m, 1H ), 1.93-1.77 (m, 1H).

^13^C NMR (CDCl_3_, 75 MHz) : δ = 137.2, 129.1, 128.2, 126.9, 48.5, 41.7, 30.4, 30.4, 25.7.

ESI - MS: m/z 211 (M+H)^+^.

**(2*R*)-1-(2-Benzyl-1,3-dithian-2-yl)-3-(benzyloxy)propan-2-ol (8):**

To a solution of **5** (7.83 g, 37.3 mmol) and **6** (2.5 g, 18.65 mmol) in dry THF (50 mL) was added a solution of *n*-BuLi in hexane (2.5 M, 14.9 mL, 37.3 mmol) at -78 °C under N_2_ for 30 min, then a solution of BF_3_·Et_2_O (1.3 mL, 9.32 mmol) in dry THF (3 mL) was added dropwise. After 1 h, the reaction was quenched by slow addition of aq sat. NH_4_Cl (15 mL). The mixture was extracted with EtOAc (3 × 20 mL). The extracts were washed with brine (2 × 10 mL), dried over anhydrous Na_2_SO_4_. Evaporation of the solvent and purification of the residue by silica gel column chromatography using 15% EtOAc-hexane as eluent afforded **8** as a yellowish oil.

Yield: (3.62 g, 64%), [α]_D_^25^ = +20 (*c* 1, CHCl_3_).

IR (KBr): 3450, 2907, 2859, 1099 cm^-1^.

^1^H NMR (CDCl_3_, 300 MHz): δ = 7.37-7.21 (m, 5H), 4.56 (s, 2H), 4.38-4.28 (m, 1H), 3.43 (d, *J* = 5.6 Hz, 2H), 3.66-3.22 (m, 3H), 3.04-2.93 (m, 2H), 2.88-2.78 (m, 2H), 2.22-2.11 (m, 2H), 2.05-1.89 (m, 2H).

^13^C NMR (CDCl_3_, 75 MHz): δ = 138, 135.2, 131.1, 128.3, 127.7, 127, 74.2, 73.2, 67.9, 52.3, 45.8, 40.6, 26.5, 26.2, 24.5.

ESI - MS: m/z 392 (M+NH_4_)^+^.

HRMS(ESI): calcd for C_21_H_30_NO_2_S_2_ (M+NH_4_)^+^: 392.1712, found: 392.1714.

**(4*R*)-5-(Benzyloxy)-4-hydroxy-1-phenylpentan-2-one (4):**

To a solution of **8** (3.61 g, 9.65 mmol ), CaCO_3_ (2.6 g, 24.12 mmol) in CH_3_CN / H_2_O (4:1, 30 mL) was added HgCl_2_ (7 g, 24.12 mmol) portionwise at room temperature. After 1 h the reaction mixture was filtered with celite. Concentration of the filtrate followed by silica gel column chromatography using 20% EtOAc-hexane as eluent afforded **4** as a colorless liquid.

Yield: (2.238 g, 82%), [α]_D_^25^ = +22.8 (*c* 1, CHCl_3_).

IR (KBr): 3450, 3029, 2907, 2863, 1710, 1099 cm^-1^.

^1^H NMR (CDCl_3_, 300 MHz): δ = 7.39-7.14 (m, 5H), 4.51 (s, 2H), 4.31-4.20 (m, 1H), 3.72 (s, 2H), 3.48-3.36 (m, 2H), 3.10 (br s, 1H), 2.76-2.59 (m, 2H).

^13^C NMR (CDCl_3_, 75 MHz): δ = 208.1, 137.8, 134.0, 129.3, 128.6, 128.4, 127.8, 127.1, 73.2, 73, 66.9, 50.7, 44.9.

ESI - MS: m/z 285 (M+H)^+^.

HRMS: calcd for C_18_H_21_O_3_ (M+H)^+^: 285.1485, found: 285.1487.

**(4*R*,6*R*)-4-Benzyl-6[(benzyloxy)methyl]-2,2-dimethyl-1,3-dioxane (3):**

To a cooled (-78 °C) stirred solution of **4** (1.1 g, 3.87 mmol) in anhydrous THF (10 mL) and MeOH (2.5 mL) under a nitrogen atmosphere was injected a solution of MeOBEt_2_ in THF (1 M, 4.4 mL, 4.64 mmol). Stirring was continued at -78 °C for 15 min before NaBH_4_ (0.165 g, 4.64 mmol) was added in one portion. After stirring at -78 °C for another 1 h, the raction was quenched (at -78 °C) by the addition of AcOH (1 mL). The mixture was poured into aqueous NaHCO_3_ solution (15 mL) and extracted with EtOAc (2 × 20 mL). The combined organic layers were washed with brine (2 × 10 mL), dried over Na_2_SO_4_, and concentrated in vacuo. The mixture fraction was purified by silica gel column chromatography using 50% EtOAc-hexane as eluent afforded **9** as a colorless oil (0.88 g, 80%). The enantiomeric excess of **9** was determined after conversion of **9** into the corresponding acetonide-protected compound **3** [2,2-dimethoxypropane (0.29 g, 4.1 mmol), CH_2_Cl_2_, catalytic amount of PPTS (pyridinium para-toluenesulfonate) (0.02 mg)] as an oil.

Yield: (0.84 g, 92%), [α]_D_^25^ = −7 (*c* 1, CHCl_3_).

IR (KBr): 1109 cm^-1^;

^1^H NMR (CDCl_3_, 300 MHz): δ = 7.37-7.16 (m, 10H), 4.58 (d, *J* = 12.3 Hz, 1H), 4.50 (d, *J* = 12.3 Hz, 1H), 4.11-3.97 (m, 2H), 3.47 (dd, *J* = 5.8, 10 Hz, 1H), 3.33 (dd, *J* = 4.7, 10 Hz, 1H), 2.94 (dd, *J* = 5.6, 13.4 Hz, 1H), 2.61(dd, *J* = 7.3, 13.4 Hz, 1H), 1.65-1.57 (m, 1H), 1.45 (s, 6H), 1.49-1.39 (m, 1H).

^13^C NMR (CDCl_3_, 75 MHz): δ = 138.2, 137.7, 129.4, 128.3, 128.2, 127.7, 127.6, 126.3, 98.7, 73.5, 73.4, 69.8, 68.5, 43.2, 33.0, 29.9, 19.6.

ESI - MS: m/z 349 (M+Na)^+^.

HRMS(ESI): calcd for C_21_H_27_O_3_ (M+H)^+^: 327.1954, found: 327.1955.

**[(4*R*, 6*R*)-6-Benzyl-2,2-dimethyl-1,3dioxan-4-yl]methanol (10):**

To a solution of lithium (0.09 g, 12.26 mmol) in liquid NH_3_ (100 mL) was added compound **3** (0.8 g, 2.45 mmol) in dry THF (10 mL) at -33 °C. The reaction mixture was stirred for 1 h at the same temperature and quenched with solid NH_4_Cl till blue color disappears. Ammonia was allowed to evaporate and the residual mixture was taken in ethyl acetate (50 mL), washed with H_2_O (2 × 20 mL) and brine (1 × 20 mL), and dried over anhydrous Na_2_SO_4_. Evaporation of the solvent and purification of the residue by silica gel column chromatography using 10% EtOAc-hexane as eluent afforded alcohol **10** as a colorless oil.

Yield: (0.52 g, 90%), [α]_D_^25^ = −11.2 (*c* 1, CHCl_3_).

IR (KBr): 3408 cm^-1^.

^1^H NMR (CDCl_3_, 300 MHz): δ = 7.30-7.12 (m, 5H), 4.08-3.96 (m, 1H), 3.94-3.83 (m, 1H), 3.59-3.48 (m, 1H), 2.97-2.87 (m, 1H), 2.65-2.54 (m, 1H), 1.87 (br s, 1H), 1.44 (s, 3H), 1.40 (s, 3H), 1.33-1.29 (m, 2H).

^13^C NMR (CDCl_3_, 75 MHz): δ = 137.6, 129.5, 128.4, 126.3, 98.8, 69.6, 66, 42.9, 31.7, 30, 19.8.

ESI - MS: m/z 259 (M+Na)^+^.

HRMS(ESI): calcd for C_14_H_20_NaO_3_ (M+Na)^+^: 259.1305, found: 259.1301.

**(3*R*,5*R*)-3-Hydroxy-5-phenylmethyldihydofuran-2-one (1):**

Dimethyl sulfoxide (0.35 g, 4.54 mmol) in CH_2_Cl_2_ (2 mL) was added to a cooled (-50 °C) and stirred solution of oxalyl chloride (0.29 g, 2.27 mmol) in CH_2_Cl_2_ (10 mL). After five min, **10** (0.45 g, 1.9 mmol) in CH_2_Cl_2_ (5 mL) was added to the mixture, followed by Et_3_N (0.964 g, 9.52 mmol). The mixture was stirred further for 30 min, brought to room temperature, H_2_O was added and stirring continued for 15 min. The organic layer was separated, the aqueous layer was extracted with CH_2_Cl_2_ (2 ×10 mL). The combined organic extracts were washed with brine (2×10 mL), dried, and concentrated in vacuo to afforded the aldhyde as colorless oil. Yield (0.422 g, 95%).

To a mixture of aldehyde (0.4 g, 1.71 mmol) in DMSO (1.6 mL) and NaH_2_PO_4_ (60 mg) in H_2_O (0.6 mL) a solution of NaClO_2_ (0.270 g, 2.395 mmol) in water (2.5 mL) was dropwise added. After stirring the reaction mixture at room temperature for 22 h, it was acidified with aqueous 3 M HCl. The reaction mixture was extracted with EtOAc (2 × 10mL), and the organic extract washed with H_2_O (2 × 10 mL) and brine (2 × 10 mL), and dried. Removal of the solvent in vacuo followed by column chromatography (silica gel, 25% EtOAc-hexane) afforded pure (*R*,*R*)-**1.**

Yield: 0.144 g (44%); white solid, mp: 77-78 ^o^C, [α]_D_^25^ = +38.1 (*c* 1, CHCl_3_).

IR (KBr): 3399, 1774 cm^-1^.

^1^H NMR (CDCl_3_, 300 MHz): δ = 7.36-7.16 (m, 5H), 4.96-4.84 (m, 1H), 4.04 (t, *J* = 7.5 Hz, 1H), 3.28 (s, 1H), 2.97 (d, *J* = 6.0 Hz, 2H), 2.40-2.16 (m, 2H).

^13^C NMR (CDCl_3_, 75 MHz): δ = 177.6, 135.2, 129.5, 128.8, 127.1, 78.3, 67, 41.07, 34.4.

ESI - MS: m/z 215 (M+Na)^+^.

HRMS(ESI): calcd for C_11_H_12_NaO_3_ (M+Na)^+^: 215.0679 found: 215.0679.

**(4*S*,6*R*)-4-Benzyl-6[(benzyloxy)methyl]-2,2-dimethyl-1,3-dioxane (12):**

To a stirred solution of tetramethyl ammonium triacetoxy borohydride (1.22 g, 4.64 mmol) in anhydrous acetone (5.0 mL) was added acetic acid (5.0 mL) and the mixture was stirred at room temperature for 30 min. The mixture was cooled to 0 °C and an acetone solution of compound **4** (1.1 g, 3.87 mmol in 2 mL of acetone) was added. The mixture was stirred at the same temperature for 3 h. The reaction was quenched with 0.5 N aqueous sodium potassium tartrate (5 mL), the mixture was diluted with CH_2_Cl_2_ (40 mL), and washed with aq sat. Na_2_CO_3_ (2 x 10 mL). The aqueous layer was back extracted with CH_2_Cl_2_ (2 x 20 mL) and the combined organic layers were washed with aq sat. Na_2_CO_3_ (2 × 20 mL). The aqueous layer was back extracted with CH_2_Cl_2_ (2 × 20 mL) and the combined organic layers were dried over Na_2_SO_4_. After concentration in vacuo, the residue was purified by column chromatography using 50% EtOAc-hexane as eluent to give a mixture of *syn* and *anti* dihydroxy compound **11** (syn/anti 1:9) and *anti* dihydroxy **11** was isolated as a colorless oil.

Yield (0.88 g, 80%).

The enantiomeric excess of **11** was determined after conversion of **11** into the corresponding acetonide-protected compound **12** [2,2-dimethoxypropane (0.29 g, 4.1 mmol), CH_2_Cl_2_, catalytic amount of PPTS (pyridinium para-toluenesulfonate) (0.02 mg)] as an oil.

Yield (0.84 g, 92%), [α]_D_^25^ = +36.1 (*c* 1, CHCl_3_).

IR (KBr): 1109 cm^-1^.

^1^H NMR (CDCl_3_, 300 MHz): δ = 7.36-7.16 (m, 10H), 4.60 (d, *J* = 12.2 Hz, 1H), 4.51(d, *J* = 12.3 Hz, 1H), 4.12-4.01 (m, 1H), 3.66 (dd, *J* = 6.4, 10.4 Hz, 1H), 3.39 (dd, *J* = 4.0, 10.38 Hz, 1H), 2.91 (dd, *J* = 6.9, 13.8 Hz, 1H), 2.67 (dd, *J* = 6.4, 14 Hz, 1H), 1.68-1.53 (m, 2H), 1.40 (s, 3H), 1.33 (s, 3H).

^13^C NMR (CDCl_3_, 75 MHz): δ = 138.1, 138.2, 129.2, 128.3, 128.2, 127.7, 127.5, 126.2, 100.5, 73.3, 72.60, 67.4, 66.2, 42.0, 34.2, 24.9

ESIMS: m/z 349 (M+Na)^+^.

HRMS(ESI): calcd for C_21_H_27_O_3_ (M+H)^+^: 327.1954, found: 327.1955.

**[(4*R*,6*S*)-6-Benzyl-2, 2-dimethyl-1, 3dioxan-4-yl] methanol (13):**

To a solution of lithium (0.09 g, 12.26 mmol) in liquid NH_3_ (100 mL) was added compound **12** (0.8 g, 2.45 mmol) in dry THF (10 mL) at -33 °C. The reaction mixture was stirred for 1 h at the same temperature and quenched with solid NH_4_Cl till blue color disappears. Ammonia was allowed to evaporate and the residual mixture was taken in ethyl acetate (50 mL), washed with water (2 × 20 mL) and brine (1 × 20 mL), and dried over anhydrous Na_2_SO_4_. Evaporation of the solvent and purification of the residue by silica gel column chromatography using 10% EtOAc-hexane as eluent afforded alcohol **13** as a colorless oil.

Yield (0.52g, 90%), [α]_D_^25^ = + 6.5 (*c* 1, CHCl_3_).

IR (KBr): 3408 cm^-1^.

^1^H NMR (CDCl_3_, 300 MHz): δ = 7.32-7.15 (m, 5H), 4.10-3.9 (m, 2H), 3.64-3.42 (m, 2H), 3-2.87 (m, 1H), 2.73-2.64 (m, 1H), 1.58 (t, *J* = 7.36, 2H), 1.39 (s, 3H), 1.31 (s, 3H).

^13^C NMR (CDCl_3_, 75 MHz): δ = 138.1, 129.1, 128.2, 126.2, 100.7, 69.3, 67.4, 65.4, 42, 33.2, 24.8, 24.7.

ESI - MS: m/z 259 (M+Na)^+^.

HRMS: calcd for C_14_H_20_NaO_3_ (M+Na)^+^: 259.1305, found: 259.1301.

**(3*R*,5*S*) -3-Hydroxy-5-Phenylmethyldihydofuran-2-one (2):**

Compound **13** (0.45 g, 1.9 mmol) was oxidized using oxalyl chloride (0.29 g, 2.27 mmol), dimethyl sulfoxide (0.35 g, 4.54 mmol) and Et_3_N (0.964 g, 9.52 mmol) in CH_2_Cl_2_ (15 mL) to give the aldehyde after work-up, as described for **1** as colorless oil.

Yield: 0.422 g (95%).

Oxidation of aldehyde (0.4 g, 1.71 mmol) with NaClO_2_ (0..270 g, 2.395 mmol) and

NaH_2_PO_4_ (50 mg) in (1.6 mL) DMSO and H_2_O (2.5 mL) followed by acidic work-up and purification furnished (3*R*,5*S*)-**2**.

Yield: 0.144 g (44%); white solid; mp: 77-78 °C.

[α]_D_^25^ = +9.2 (*c* 1, CHCl_3_)

IR (KBr): 3411, 1775 cm^-1^.

^1^H NMR (CDCl_3_, 300 MHz): δ = 7.36-7.17 (m, 5H), 4.63-4.47 (m, 2H), 3.52 (s, 1H), 3.17-3.09 (m, 1H), 2.99-2.95 (m, 1H), 2.66-2.55 (m, 1H), 2.04-1.88 (m, 1H).

^13^C NMR (CDCl_3_, 75 MHz): δ = 177.4, 135.4, 129.5, 128.7, 78.3, 68.5, 41.2, 36.5.

ESI - MS: m/z 215 (M+Na)^+^.

HRMS: calcd for C_11_H_12_NaO_3_ (M+Na)^+^: 215.0679 found: 215.0679.

Note: Copies of IR, ^1^H NMR and ^13^C NMR of **(3*R*,5*R*)-1** and **(3*R*,5*S*)-2** were provided.


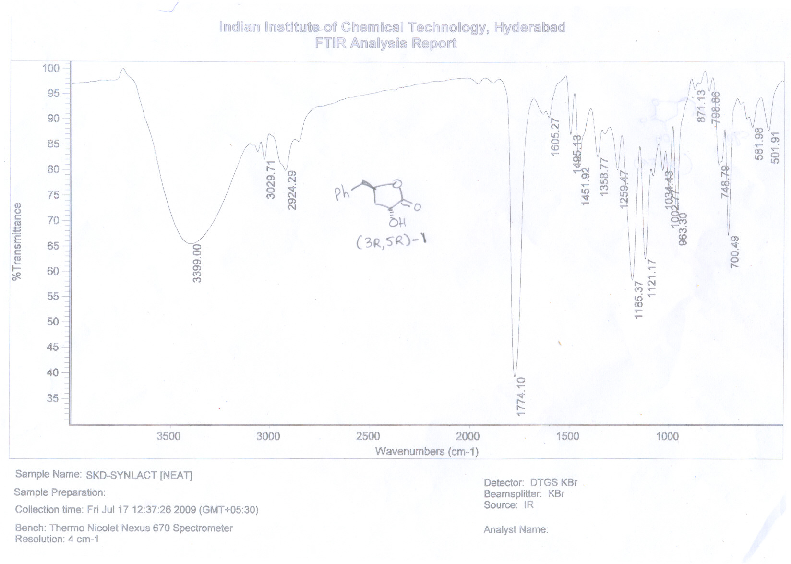


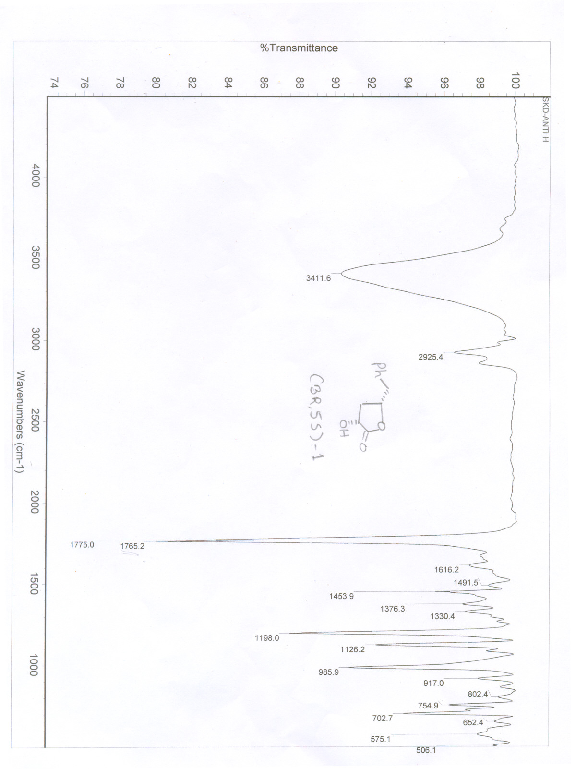

Supplement: File 1 — Experimental section and analytical data. [file Beilstein_J_Org_Chem-06-08-s001.doc]
